# Development Process of a Clinical Decision Support System for Empiric Antibiotic Therapies in Patients With Sepsis: Case Study

**DOI:** 10.2196/79929

**Published:** 2026-05-13

**Authors:** Sophie Schmiegel, Hannah Marchi, Philipp Hege, Svenja Elkenkamp, Juliane Duevel, Christoph Düsing, Wolfgang Greiner, Sean Selim Scholz, Dominic Witzke, Johannes J Tebbe, Michael Wehmeier, Olaf Kaup, Rainer Borgstedt, Sebastian Rehberg, Philipp Cimiano, Christiane Fuchs

**Affiliations:** 1 Data Science Group, Faculty of Business Administration and Economics, Bielefeld University, Universitätsstraße 25, North Rhine-Westphalia, Bielefeld, 33615, Germany, 49 521 106 2576; 2 AG 5 - Health Economics and Health Care Management, School of Public Health, Bielefeld University, Bielefeld, Germany; 3 Centre for electronic Public Health Research (CePHR),, School of Public Health, Bielefeld University, Bielefeld, Germany; 4 Center for Cognitive Interaction Technology (CITEC), Faculty of Technology, Bielefeld University, Bielefeld, Germany; 5 Department of Anaesthesiology, Intensive Care, Emergency Medicine, Transfusion Medicine and Pain Therapy, Medical School and University Medical Center OWL, Bielefeld University, Protestant Hospital of the Bethel Foundation, Bielefeld, Germany; 6 Division of Internal Medicine, Department of Gastroenterology and Infectiology, Medical School and University Medical Center OWL, Bielefeld University, Klinikum Lippe, Detmold, Germany; 7 Institute of Laboratory Medicine, Microbiology and Transfusion Medicine, Klinikum Bielefeld, Bielefeld, Germany; 8 Institute of Computational Biology, Computational Health Center, Helmholtz Munich, Oberschleißheim, Germany

**Keywords:** antibiotic prescription, antibiotic resistances, clinical decision support system, multiclass classification, sepsis, time series classification

## Abstract

**Background:**

Antibiotic therapies are the main treatment for bacterial infections, but growing antibiotic resistance is a major global health threat, severely impacting patients with sepsis. Rapid selection of the most effective antibiotic therapy is critical for survival and for preventing further resistance. Physicians must consider numerous factors for proper empiric treatment selection. A clinical decision support system (CDSS) aims to support physicians in this process, facilitating rapid and targeted therapy.

**Objective:**

The purpose of this work is to explore the extent to which the realization of a CDSS is possible based on the data available to us and to document insights gained during the development of a foundational model designed to assist physicians in determining empiric treatment options for patients with sepsis. In this regard, rather than aiming to develop a CDSS for clinical application, we highlight the importance of close interprofessional collaboration between scientists from various disciplines and analyze the effects of data quality and quantity on the performance of our statistical models.

**Methods:**

Empirical scientists conducted interviews with medical practitioners to acquire the medical knowledge required to develop sound statistical models. We developed and applied 2-step cross-sectional, as well as time-series classification models, to carefully preprocessed data of patients with sepsis admitted to the intensive care unit of a German hospital.

**Results:**

We identified several factors as crucial information for valid decisions on empiric therapy for treating patients with sepsis. These include the patients’ core data, especially the infection focus. To prevent further resistance, individual risk factors such as travel history and professional background should be considered. The evaluation of a therapy’s effectiveness is mainly based on the patient’s general condition and blood values such as procalcitonin and interleukin 6. One key factor in the acceptance of a CDSS is the explainability of the results produced by the applied methods. Our models demonstrated mainly weak predictive ability for all considered empiric antibiotic therapies. However, they are not yet suitable for use in clinical practice, especially as they are based on prescribing habits rather than on optimal treatment decisions.

**Conclusions:**

This work highlights the importance of interprofessional collaboration between medical experts and model developers, ensuring that data quality and clinical relevance are central to the process. It emphasizes the urgent need for high-quality, comprehensive data to overcome challenges such as data discontinuity and improve model performance, particularly through enhanced digitization in health care. This feasibility study will facilitate future efforts to develop a CDSS for treating patients with sepsis and to translate it into clinical use.

## Introduction

### Motivation

Antibiotic therapies represent a milestone within pharmacological and clinical research and are commonly used in the treatment of bacterial infections ranging from mild cystitis to life-threatening sepsis. Sepsis is a severe and rapidly progressing course of a bacterial infection that needs to be treated within the first 3 hours after diagnosis to increase the chances of patient survival [1]. The chance of treatment success rises with increased coverage of pathogens; thus, the Surviving Sepsis Campaign Guidelines for Management of Sepsis and Septic Shock 2021 [1] recommend a broad-spectrum antibiotic as empiric (ie, the initial) therapy. Accordingly, German hospital guidelines recommend the use of a broad-spectrum antibiotic, in particular if the focus of infection is unknown. While broad-spectrum antibiotics are likely to be effective, they come with a variety of side effects, such as gastrointestinal complaints and drug fever [2], and harbor the risk of developing antibiotic resistance [3,4]. Such resistance poses a global threat to health care systems [5,6]. Thus, an important goal is to minimize the use of broad-spectrum antibiotics. A direct point of action for this is inappropriate prescriptions, for example, in the case of viral infections [7], and still common self-prescriptions due to low-threshold access to antibiotics [8]. Overall, preventing the development of further resistance requires consistent antibiotic stewardship and support in selecting targeted antibiotic therapies. The latter is particularly difficult in the case of severe disorders such as sepsis, which are the focus of this work. Even for experienced physicians, the diagnosis and treatment of sepsis are challenging tasks [9].

### Background

#### Project Background

This work has emerged from and shows an excerpt of the research in the interprofessional project “KINBIOTICS” [10], funded by the German Federal Ministry of Health from 2020 to 2024. An overarching objective of the “KINBIOTICS” consortium project was to develop an artificial intelligence (AI)–based clinical decision support system (CDSS) for the individualized prediction of targeted, effective antibiotic therapies with low side effects for patients with sepsis. Such a CDSS would help clinicians make rapid and targeted treatment decisions and thus save lives and reduce the progressive occurrence of antimicrobial resistance. Additionally, the process of developing a data-driven CDSS has the potential to reveal previously unconsidered patterns in the data, thereby providing substantial benefits to the current prescription process.

Research in the “KINBIOTICS” project was conducted by medical practitioners and empirical scientists from various disciplines, including information technology, data science, biotechnology, health sciences, and medicine. The scientists were affiliated with 5 institutions, namely Bielefeld University, University of Siegen, Evangelisches Klinikum Bethel, Klinikum Bielefeld, and Klinikum Lippe. The project was organized in work packages addressing a range of topics, including the prediction of the pathogen and resistance spectrum by nanopore sequencing [11], the setup of a data warehouse including the creation of preconditions for federated learning [12], the development of a resistance observatory [13], evaluation of a pilot CDSS [14], statistical model building as a basis for the CDSS, and the support system’s integration into clinical routine [15].

To achieve the project goals, model constructors and medical professionals were in close dialogue with each other, and this turned out to be particularly helpful for the process of data selection, data preprocessing, and statistical model fitting. In the following, we focus on this process and the steps we found essential to be taken before the development of an AI-based CDSS.

#### Sepsis

Sepsis is one of the main causes of illness and death all over the world. In 2017, an estimated 48.9 million incident cases of sepsis were recorded worldwide, and 11.0 million sepsis-related deaths were reported, representing 19.7% of all global deaths [16]. Therefore, the World Health Organization (WHO) urged its members through a resolution to include prevention, diagnosis, and treatment of sepsis in national health care systems and to develop and implement standard and optimal care for diagnosing and managing sepsis in health emergencies [17].

Sepsis has been defined as a life-threatening organ dysfunction caused by a dysregulated host response to infection (Sepsis-3 definition) [18]. The infection can be caused by most types of microorganisms, including bacteria, fungi, viruses, and parasites [19]. Organ dysfunction is characterized using the sequential organ failure assessment (SOFA) score [20], including 6 organ systems (respiratory, coagulation, liver, cardiovascular, central nervous system, and renal) with a rise of at least 2 points following the infection. The most severe state of sepsis is defined as septic shock with persistent hypotension requiring vasopressor therapy and a serum lactate level of more than 2 mmol/L despite adequate volume resuscitation. This status is characterized by circulatory, cellular, and metabolic abnormalities leading to an increased mortality of up to 40% [18].

According to the resolution of the WHO, international scientific organizations started the Surviving Sepsis Campaign in 2002, developing guidelines for the treatment of sepsis and septic shock [1]. According to these guidelines, sepsis and septic shock are medical emergencies requiring rapid diagnosis and immediate treatment. Consequently, the following measures should be carried out as quickly as possible: measurement of the lactate level, antibiotic susceptibility testing, administration of broad-spectrum antibiotics, initiation of appropriate fluid administration and vasopressors if necessary, and remeasurement of the lactate level. In particular, the administration of antibiotics should not be delayed or fail to target the pathogen. The rationale behind these measures is that the time until effective antimicrobial therapy is a crucial determinant of survival in septic shock [21]: a delay in the initiation of effective antimicrobial therapy is associated with a mean decrease in survival of 7.6% per hour.

In clinical practice, the microbiological proof of the pathogen causing sepsis or septic shock is most often missing at the time of diagnosis. Therefore, the initial administration of antibiotics is empiric and generally follows the Tarragona strategy first described by Sandiumenge et al [22]. The principles of this therapy involve assessing patients and their risk factors to “hit hard and early”, aiming at an immediate administration of an effective dose of antibiotics while considering the local antimicrobial situation. It also includes accurately identifying the site of infection and understanding the pharmacodynamics and pharmacokinetics of the antibiotics. Furthermore, it is essential to remain focused by continually reevaluating the effectiveness of administered antibiotics. Finally, the treatment should be de-escalated accordingly after identifying the pathogen.

### Focus of This Work

Many disciplines aim to improve antibiotic therapies, including microbiology, pharmacology, chemistry, and clinical research. Research and knowledge transfer can be significantly more effective when they operate across disciplinary boundaries and connect fundamental research with clinical practice. In our work, we specifically focus on the interprofessional work between scientists from various disciplines with empirical and clinical backgrounds on the way to a CDSS for antibiotic treatment, which is an information system designed to assist (but not replace) physicians in making empiric treatment decisions for patients with sepsis. The particular value of our contribution arises from the close collaboration between disciplines and the integration of guidelines, constraints, and clinical experience into data analysis. We refrain from pursuing the development of a CDSS for widespread use in clinical practice. Instead, the paper constitutes a feasibility study and consolidates insights obtained during the development process to provide guidance for projects with comparable objectives. We describe preparatory steps required before the construction of statistical models used for a CDSS and model physicians’ decisions regarding empiric antibiotic therapy. In this regard, we highlight the relevance of high data quality and close exchange between medical experts and model constructors.

### State of Research

The causes, diagnosis, treatment, and consequences of sepsis are highly relevant in various fields of research. In line with the focus of our work, machine learning and deep learning have commonly been applied to analyze data of patients with sepsis. In particular, numerous studies cover the early and accurate prediction of the onset of sepsis, for example [23-27], and Moor et al [28] provide a systematic literature review for early prediction of sepsis in intensive care units (ICUs). Further, many studies pursue the goal of predicting antimicrobial resistance. The literature review by [29] identified 12 studies that use machine learning in the context of antibiotic susceptibility pattern prediction (eg, [30-32]). Additionally, Sakagianni et al [29] selected 8 studies on machine learning–assisted antibiotic resistance prediction (eg, [33-35]). Paul et al [36] developed a computerized decision support system to predict appropriate antibiotic therapies to avoid the use of broad-spectrum antibiotics for treatment of infectious diseases. This study also considered the benefits of antibiotics (eg, reduction in the length of hospital stay) as well as costs (eg, adverse events). Such CDSS may also be beneficial for patients with sepsis, in particular in assisting physicians in selecting a proper empiric antibiotic therapy.

## Methods

### Data Acquisition and Preprocessing

#### Medical Knowledge Compilation

As described in the Focus of This Work subheading, we attributed great importance to interprofessional collaboration to appropriately meet the challenges of adequate treatment of sepsis, a multifaceted complication of an infection. Consequently, medical practitioners and empirical scientists maintained continuous dialogue to ensure a deep and comprehensive understanding of both the medical processes and the methodological requirements. This knowledge exchange aimed to guarantee that decisions made during data preprocessing and statistical model construction were congruent with the clinical procedure.

On the operational level, the empirical scientists conducted a series of interviews with predefined questions with representatives of the 3 participating hospitals, including physicians working at the bedside of patients with sepsis. In this way, nonclinicians were given a comprehensive overview of treatment options and guidelines, and relevant stakeholders were engaged at all stages of the CDSS development process.

**Figure 1.**
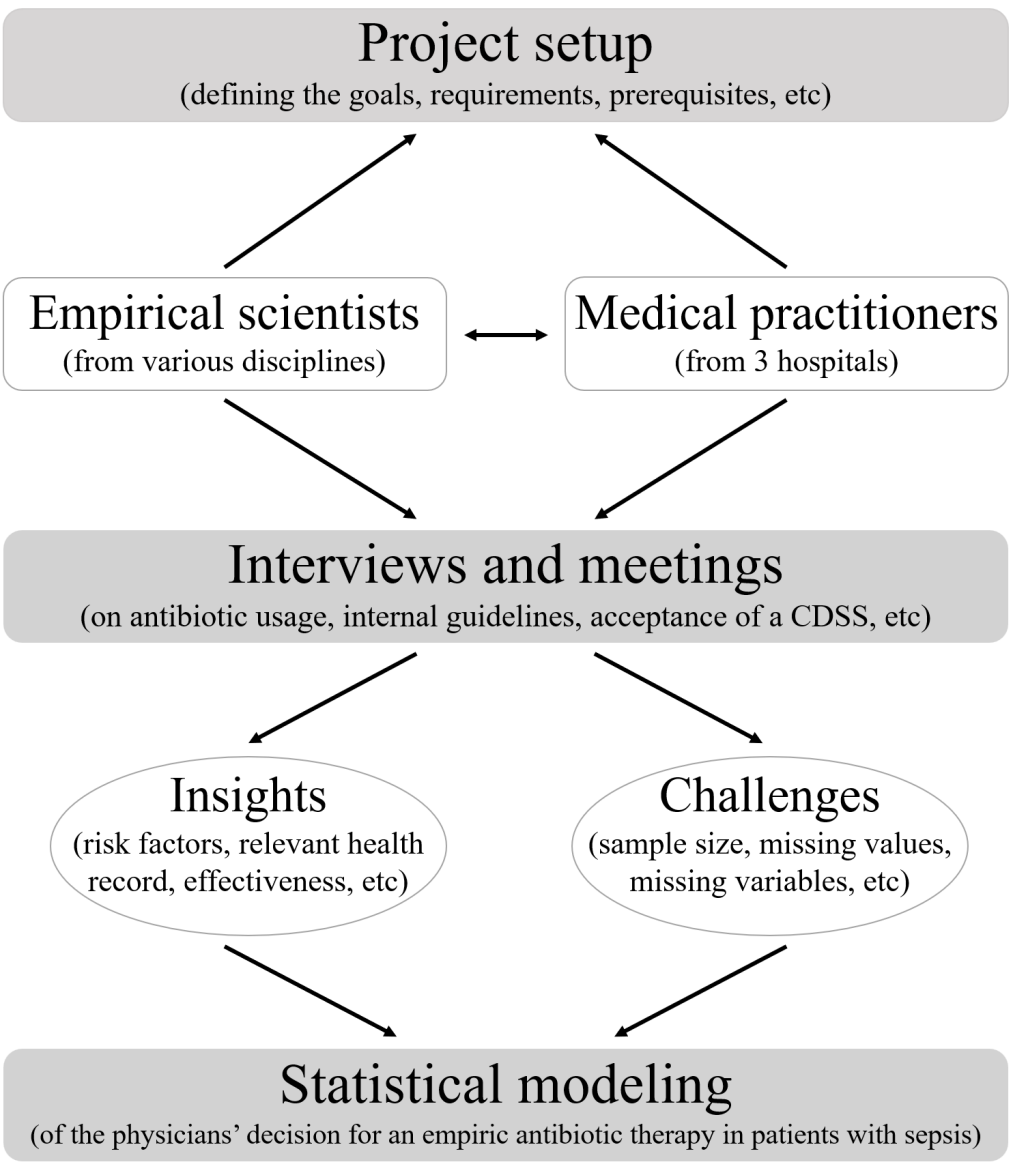
Schematic representation of the process within the “KINBIOTICS” project aimed at modeling physicians’ decision-making for empiric antibiotic therapy in patients with sepsis. CDSS: clinical decision support system.

In an initial interview series, 2 aspects were of primary interest: first, the nature and use of broad-spectrum antibiotics, along with aspects of combination therapies; second, the evaluation process for the effectiveness of administered therapies and the critical factors that influenced this evaluation. In 2 further series of interviews, we focused on relevant determinants of successful implementation of a CDSS in clinical routine settings by assessing the clinicians’ general acceptance of such a system and identifying necessary information to be provided to make it as trustworthy as possible. In addition to the interviews, we conducted biweekly meetings between representatives from the hospitals and data scientists. Further, all 3 hospitals provided their internal guidelines for antibiotic administration and dosage depending on diagnosis and identified pathogens. This facilitated the data scientists’ understanding of the specific procedures and decision-making processes. Figure 1 illustrates the overall project process, with a focus on statistical model development.

#### Data

We use retrospective health records from patients admitted to the ICU of the German hospital Evangelisches Klinikum Bethel between 2012 and 2023. All patients in this dataset were admitted to an intensive or intermediate care unit and diagnosed with sepsis or septic shock (in particular, they were diagnosed with one of the *ICD-10* (*International Statistical Classification of Diseases, Tenth Revision*) [37] codes: A40.0, A40.1, A40.2, A40.3, A40.8, A40.9, A41.0, A41.1, A41.2, A41.3, A41.4, A41.51, A41.52, A41.58, A41.8, A41.9, R57.2, or R65.1. The data were derived from 3 sources: first, the clinical information system included time-constant core data and diagnoses, as well as blood values and vital signs measured over the period of hospitalization. Second, the patient data management system contained the administration of catecholamines and antibiotics over time. Third, information about microbiology analyses was available from the hospital’s laboratory, representing results of antibiotic susceptibility testing based on patient samples such as blood, urine, or smear. However, data on administered catecholamines and microbiological analyses were not included in the analyses, as microbiology data, in particular, were not available at the time of diagnosis.

Before data analysis, we carefully preprocessed the data. As a first step, we excluded patients who did not receive any antibiotic therapy or who had not yet reached the age of 18 years at the time of admission. In collaboration with medical experts, erroneous values in variables such as age, height, and weight were either adjusted or removed, and thresholds for blood values and vital signs were included in accordance with medical recommendations. The suspected focus of infection was not documented in the available data. However, given its potential importance for addressing the research question, we derived the suspected infection focus from patients’ *ICD-10* codes. To this end, a medical expert categorized all sepsis-related *ICD-10* codes from the clinical information system database according to their infection focus. This procedure resulted in many patients having more than one suspected infection focus. Consequently, we split the foci per patient into several variables, taking into account the order of occurrence. For further analysis, however, we exclusively used the first focus and refer to it as the suspected infection focus on the remainder of this text.

A complication occurred because the time point of diagnosis was only insufficiently documented in the patients’ records. Consequently, we assumed that the time of sepsis diagnosis corresponded to the time of the initial antibiotic administration; this serves as an approximation that appears to be adequate to fulfill the objective of this work. Additionally, some patients were represented several times in the dataset. They were either repeatedly admitted to the hospital or had multiple infections during one hospital stay. In particular, there were 2482 individual patients with a total of 2986 infections, resulting from up to 6 infections per patient. We divided one hospital stay into 2 separate stays when no antibiotic was administered for at least 5 days, and there was a change in the infection focus during that time. Henceforth, for simplicity, we use the term “patients” in place of “infections”, even in cases where a single patient is represented by multiple infections in the dataset.

**Figure 2.**
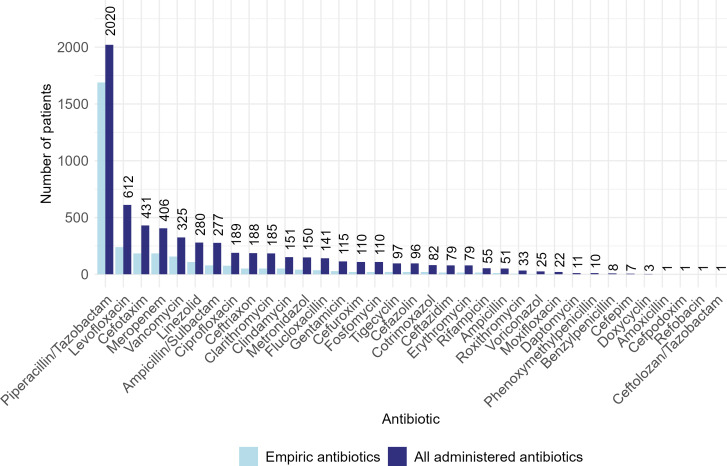
Number of patients who received the respective antibiotics between 2012 and 2023. The light blue bars represent all empiric antibiotic prescriptions (simultaneously administered therapies, ie, combined therapies, are shown separately), while the dark blue bars additionally include all subsequent administered antibiotics. If antibiotics were administered more than once to one patient, they are counted only once. Printed numbers refer to the dark blue bars.

The obtained dataset is presented as follows: core data were available for 2986 patients and contained 26 variables. The age of the patients ranged from 18 to 99 years, with an average age of 67.56 years; approximately 36% (n=1067) of patients were female. A total of 1257 (42%) of all patients died during the hospital stay. The aforementioned categorization to derive the suspected infection focus ultimately yielded the following 16 categories (with the number of occurrences in brackets): lungs (n=1074); urogenital tract (n=498); gastrointestinal tract (n=353); prosthesis inflammation (n=175); skin or muscle (n=118); bile (n=101); brain (n=82); bones, joints, tendons, or intervertebral discs (n=60); heart (n=34); vessels (n=14); liver (n=11); ear, nose, or throat (n=10); esophagus (n=8); oral cavity (n=3); spleen (n=1); and eye (n=1). For all patients, time series of vital signs and blood values (31 variables) were available from a maximum of 88 days before and up to 1610 days after the date of diagnosis. However, the measurement and documentation of time series variables were carried out with varying quantities and frequencies. Table 1 lists all available variables. Diagnoses were documented for 2945 patients with 3319 unique *ICD-10* codes in total; 24 of these were connected to sepsis or septic shock. In addition to these *ICD-10* codes, we had information about whether a patient had diabetes or required dialysis; further information about possible comorbidities was not available. Further, the data included information about administered antibiotics, including drug names as well as therapy start and end dates, for all patients. A total of 35 distinct antibiotics were administered at least once. We define the first antibiotic administered to a patient (during diagnosis or afterwards) as empiric. An overview of all antibiotics and their frequencies is provided in Figure 2.

**Table 1.** Description of the variables contained in the preprocessed dataset.

Source and variable	Unit	Description
Core data		
Infection ID	—^a^	Unique identifier per infection and patient
Admission date	Date	—
Discharge date	Date	—
Suspected time of diagnosis	Date	Documented date or time point of first administration of antibiotic treatment
Age	Years	—
Sex	—	Male or female
Diabetes	—	Patient has diabetes (“yes” or “no”)
Death	—	Patient died during hospital stay (“yes” or “no”)
Weight	kg	—
Height	cm	—
BMI	kg/m^2^	—
Suspected infection focus	—	Suspected source of the infection (16 different categories)
Time series data		
Infection ID	—	Unique identifier per infection and patient
Date of record	Date	—
Minutes after diagnosis	Minutes	Difference between the time of diagnosis and the time of record
SOFA^b^ score	—	SOFA score (containing FiO_2_^c^/pO_2_^d^, thrombocytes, bilirubin, mean arterial pressure, GCS^e^ score, and creatinine), ranging from 0 to 24
GCS	—	GCS score ranging from 3 to 15
RASS^f^	—	RASS score ranging from –5 to 4
Dialysis	—	Patient on dialysis (“yes” or “no”)
Ventilation	—	Patient is ventilated (“yes” or “no”)
Heart rate	bpm	—
Body temperature	°C	—
Respiratory rate	breaths/min	—
Urine	mL/h	—
Diastolic Ibp	mm Hg	Invasive diastolic blood pressure
mIbp	mm Hg	Invasive mean blood pressure
Systolic Ibp	mm Hg	Invasive systolic blood pressure
Diastolic NIbp	mm Hg	Noninvasive diastolic blood pressure
mNIbp	mm Hg	Noninvasive mean blood pressure
Systolic NIbp	mm Hg	Noninvasive systolic blood pressure
CAM-ICU	—	Confusion Assessment Method for the Intensive Care Unit (delirium “yes” or “no”)
Bilirubin	mg/dL	—
FiO_2_	%	Fraction of inspired oxygen
pO_2_	mm Hg	Partial pressure of oxygen
Leukocytes	/nL	Number of white blood cells in the blood
Leukocytes in the liquor	/µL	Number of white blood cells in the cerebrospinal fluid
Thrombocytes	10^3^*/*mm^3^	Number of thrombocytes in the blood
CRP	mg/L	C-reactive protein
PCT	ng/mL	Procalcitonin
GFR	mL/min	Glomerular filtration rate
Creatinine	mg/dL	—
IL-6	pg/mL	Interleukin 6
Lactate	mmol/L	—
Blood sugar	mg/dL	—
Oxygenation index	mm Hg	—
Antibiotic	—	Name of administered antibiotic therapy
Diagnosis	—	*ICD-10^g^* codes of diagnosis

a Not available.

b SOFA: Sequential Organ Failure Assessment.

c FiO_2_: fraction of inspired oxygen.

d pO_2_: partial pressure of oxygen.

e GCS: Glasgow Coma Scale.

f RASS: Richmond Agitation-Sedation Scale.

g *ICD-10*: *International Statistical Classification of Diseases, Tenth Revision*.

A review of the core data reveals that the variables weight, height, and BMI each contain 57% missing values. In addition, 15% (n=443) of patients have no suspected infection focus. However, all other information from the core data are completely available for all patients.

For a more effective comparison between patients, we extend the time series data containing laboratory values and vital signs to include the variable “minutes after diagnosis.” This is calculated as the difference between the suspected time of diagnosis and the time of record. Importantly, not all variables are measured constantly or at equidistant intervals; certain values, such as heart rate, however, are frequently measured. Conversely, blood values, including lactate and C-reactive protein (CRP), are typically assessed no more than once per day.

In summary, when considering the average availability of measurements per patient (excluding those with no recorded measurements for a specific value), the values with the lowest frequency of measurement are leukocytes in the liquor, with an average of 0.87 measurements per patient, and procalcitonin, with an average of 4.10 measurements per patient. In contrast, along with dialysis and ventilation, heart rate (mean of 177.31 measurements per patient) and invasive blood pressure (mean of 160.87 systolic measurements per patient) are the values with the highest availability. An overview of the frequency and availability of all time series values is shown in Multimedia Appendix 1.

### Statistical Analysis

#### Overview

On our way to a CDSS applicable in situations where patients have just been diagnosed with sepsis, we aimed, as an intermediate step, at statistical models that are able to predict an empiric antibiotic therapy. Consequently, we intend to predict the prescribed empiric antibiotic therapy based on patients’ core data and health records. We address this goal through statistical classification, where each distinct therapy defines one class, and we train a classifier that assigns class memberships to patients. This is possible since the dataset used for training contains the relevant information about the administered initial antibiotic therapy, that is, the true class membership.

To start with, we assume a maximum of one measurement per variable for newly diagnosed patients. This mimics the constraints for a rapid decision after sepsis onset. Accordingly, in the Cross-Sectional Classification subheading, we reduce our dataset to cross-sectional data and apply a cross-sectional classification model (CCM).

In addition, we ask the question of how recommendations could have been improved when based on time series data. To that end, we consider time series classification models (TSCMs) in the Time Series Classification subheading.

#### Cross-Sectional Classification

Classification is a statistical method for supervised learning, where the true class memberships of training samples are known and can be used during model training to learn a mapping of covariates to classes. After model training, this learned mapping is applied to new data to make predictions for each sample. In our work, we rely on the following classifiers, which are robust to multicollinearity: random forests (RFs), gradient boosting classifiers (GBCs), support vector classifiers (SVCs), and multilayer perceptrons (MLPs).

To train the CCMs, we further prepare the data additionally to the general preprocessing. In particular, we generate covariates that contain cross-sectional instead of time series information as required: for each time-resolved variable, we obtain the relevant values for all patients by selecting the first value measured within the first hour after diagnosis, motivated by the clinical practice to obtain key measurements immediately after diagnosis to capture the current status of a patient. We assume this information to be available at the time of treatment decision. If no measurement is available within this time interval, we use the most recent value within 24 hours before the sepsis diagnosis. Further, we summarize categories of the suspected infection focus occurring less than 50 times as “others.”

Many variables in the data have a high proportion of missing values. We refrain from data imputation, for example, multiple imputation [38] or filling missing values by the mean across all samples, as we want to avoid introducing additional uncertainty into the data. Instead, we discard those variables that have 30% or more missing values. Accordingly, we keep the following variables: age, sex, heart rate, average arterial blood pressure, body temperature, fraction of inspired oxygen, pressure of oxygen, leukocytes, thrombocytes, CRP, creatinine, lactate, blood sugar, bilirubin, requiring dialysis, requiring ventilation, and suspected infection focus. These serve as covariates for the CCMs. We do not include systolic and diastolic blood pressure, SOFA, oxygenation index, or glomerular filtration rate as this information is already contained in other variables. After variable selection, we filter for complete cases (ie, patients without any missing values) and end up with 1194 patients for model estimation.

As the target variable (ie, the variable that determines a patient’s class membership), we consider the first administered antibiotic therapy (or a combination of several simultaneously administered therapies) for a patient, that is, the antibiotic treatment within the first hour after the diagnosis.

In the resulting dataset, we are faced with highly imbalanced class sizes. We mitigate this imbalance by merging antibiotic therapies with fewer than 20 occurrences as “others.” This avoids the CCMs being trained on too few samples for some antibiotics, but even subsequent to this step, imbalanced classes in the target variable remain evident (Table 2): while the largest class, “Piperacillin/tazobactam,” contains 550 patients, the smallest class, namely a combined therapy of “Piperacillin/tazobactam” and “Levofloxacin,” consists of only 21 patients.

**Table 2.** Numbers of antibiotic therapies in the German hospital cohort used for the 2-step models.

Antibiotic therapy	Count
Piperacillin/Tazobactam	550
Other therapies	174
Cefotaxime	104
Meropenem	77
Ampicillin/sulbactam	75
Levofloxacin	54
Ceftriaxone	44
Cefuroxime	33
Vancomycin	33
Ciprofloxacin	29
Piperacillin/tazobactam–levofloxacin	21

Common rebalancing techniques, such as downsampling, would lead to a substantial decrease in the sample size. Thus, instead, we divide the original classification task into 2 consecutive tasks: in a first step, we perform binary classification to predict the majority class “Piperacillin/tazobactam” vs all others; in a second step, we perform multiclass classification to further distinguish between the remaining antibiotic therapies. As described in this section, we apply RF, GBC, SVC, and MLP as classifiers at both steps.

To account for the fairly small amount of data and to avoid model overfitting, we use nested cross-validation (CV) with an inner and an outer loop [39]. The outer loop consists of 10 folds and splits the whole dataset into 10 disjunct sets. The inner loop consists of 5 folds, allowing for a grid search to optimize the models’ hyperparameters. For RF, we tune the splitting criterion, the total number of fitted trees, the maximum depth of the trees, and the minimum number of samples to perform a subsequent split. For GBC, in addition to the latter 2 parameters, we optimize the learning rate as well as the number of boosting stages. For SVC, the regularization parameter *C*, the kernel type, and the degree in case of a polynomial kernel are tuned during the grid search. For MLP, we optimize the number of hidden layers assuming each layer consists of 100 neurons, the activation function, the solver, the *L2* regularization term, and the initial learning rate. To measure the models’ performance, we use the *F*_1_-score, which is the harmonic mean of precision and sensitivity.

Estimating the binary model does not require any rebalancing. The dataset (before applying CV) contains 550 patients receiving “Piperacillin/tazobactam” (Table 2) and 644 patients receiving a different therapy. This is, however, different for the multiclass classification in the second step. The model is trained on those patients from the training set who received an antibiotic therapy other than “Piperacillin/tazobactam.” As shown in Table 2, there are still large differences between the remaining 10 class sizes. At this point, after excluding the most dominant class “Piperacillin/tazobactam,” we opt for rebalancing to prevent the model from ignoring the minority classes. As the difference between the largest and smallest class sizes is substantial, we downsample the classes whose sizes exceed the average frequency in the training data for the second step and upsample the other classes accordingly. This procedure prevents the dataset from becoming too small, which at the same time avoids excessive duplication of samples.

#### Time Series Classification

We apply a TSCM to investigate whether more information is beneficial to correctly predict an empiric antibiotic therapy for patients with sepsis. In contrast to the models described in the Cross-Sectional Classification subheading, TSCMs consider a time series of a sample instead of using just one measurement per variable. In the medical context, this poses a particular challenge, given that patients’ health records are typically measured at different time points rather than at uniform intervals. Consequently, the individual time series often vary in length. This requires techniques that can deal with unequal sequences, such as the *k*-nearest neighbor (*k*-NN) algorithm using dynamic time warping (DTW) to measure distances.

To apply *k*-NN with DTW to our data, we consider the patients’ time series starting at 24 hours before sepsis diagnosis and up to 1 hour afterwards. Our approach is based on the fact that no later measurements are available for new patients due to the need for fast antibiotic treatment. We use the same covariates and target variable as for the CCMs described in the Cross-Sectional Classification subheading. In contrast to these models, we cannot exclusively use complete cases for the TSCM, as the data basis would become far too small. Therefore, we use forward or, if no previous value is available, backward propagation to fill the missing values in the time series. This procedure is consistent with clinical practice: measurements are considered valid for 24 hours as long as the treating physicians have not seen a need for new values, for example, in case of a deterioration in a patient’s health condition. The procedure to estimate the models is the same as for the CCMs: in a first step, we perform binary classification to derive a probability for “Piperacillin/tazobactam,” and in a second step, we aim at classifying those antibiotic therapies that are different from “Piperacillin/tazobactam.” Again, we use a nested CV and a combined procedure of upsampling and downsampling for class rebalancing in the second step.

### Ethical Considerations

Ethical approval was obtained from the Ethics Committee of the Westphalia-Lippe Medical Association and the University of Münster (Ethik-Kommission der Ärztekammer Westfalen-Lippe und der Westfälischen Wilhelms-Universität Münster; case no 2021‐699-f-S).

## Results

### Requirements and Insights From Clinical Knowledge

#### Overview

To enhance the reliability of the CDSS and to acknowledge its limitations, we investigated before the data analysis which kinds of information were regarded as relevant from a clinical perspective. A purely data-driven approach, in contrast, would have searched the wide variety of variables for patterns in an unrestricted manner. However, this approach carries the risk of inducing spurious correlations or therapies that may turn out impractical. Through the interviews and discussions described in the Medical Knowledge Compilation subheading, the following facts and requirements were compiled and subsequently served as a knowledge base for the nonclinical scientists in this project. The Challenges From Data Availability subheading describes to what extent the requirements were met by our data, including which variables were unavailable.

#### Including Relevant Factors for Treatment Decision

Following a diagnosis of sepsis, there are multiple factors that the treating physician takes into consideration when selecting an appropriate antibiotic. These include, among others, core data, measured vital signs and blood values, patient allergies, known comorbidities, adverse effects, and image data (eg, x-ray images or computer tomography scans). Further, the identification of the infection focus is of utmost importance as it allows for the identification of potential pathogens once the affected organ is confirmed. However, in some cases, the focus is not provided as it is not directly recognizable.

#### Considering Risk Factors for Antibiotic Resistances

To take potential resistances into account, whether on a global or patient-specific basis, it is necessary to consider additional kinds of information: the patient’s origin and professional background, as well as their travel history, previous hospitalization or stay in nursing homes, and antibiotic intake within the last 3 to 6 months. This information may offer insights into the present pathogens and suspected resistances for specific patients.

#### Evaluation of Antibiotic Effectiveness

With regard to the evaluation of antibiotic effectiveness (ie, the assessment of whether the administration of a specific antibiotic was successful in treating a particular case of sepsis), the most important information is the SOFA score, oxygenation index, procalcitonin, CRP, interleukin 6 (IL-6), body temperature, and circulatory parameters such as blood pressure, heart rate, and respiratory rate. From these, especially procalcitonin and IL-6 are also identified in the literature [40]. Furthermore, leukocytes, thrombocytes, bilirubin, creatinine, lactate, the Confusion Assessment Method for the Intensive Care Unit, and urine output are considered as potential covariates. Generally, for an effective therapy, laboratory values should show a clear improvement. However, the clinical experts emphasized that the identification of relevant factors depends on the infected organ. Furthermore, they highlighted that the general health condition of a patient is more important than laboratory values. If a patient’s well-being improves, irrespective of laboratory values, and they demonstrate, for example, increased responsiveness and alertness, it can be assumed that the administered therapy was effective.

#### Expectations and Acceptance of CDSS

One of the conducted interview series addressed the physicians’ opinion on the practical implementation of a CDSS and revealed high interest in and need for support from such a system. This is because clinicians must process the available information within a short time frame, often facing incomplete data when the critical health status of a patient necessitates a rapid therapeutic decision. Furthermore, increasing antimicrobial resistance demands careful consideration of the numerous alternative therapies available. The establishment of a support system, therefore, is of great added value for physicians with limited practical experience, thereby helping them to make well-informed treatment decisions. However, it was also noted that there are still many operational hurdles to overcome, such as the lack of digitization in clinics or the availability of reliable and reasonable retrospective data, which hinder its use in practical hospital settings. A detailed analysis of the interviews is provided by Düvel et al [14]. Another important requirement for a CDSS, highlighted by the medical professionals, concerned the interpretability of the underlying statistical models. Since we use interpretable classification methods rather than AI tools here, this constraint is met, but must be given careful consideration in general.

### Challenges From Data Availability

A goal of this study was to determine the extent to which the ideal and theoretically formulated requirements for a CDSS align with the reality of clinical practice. The result of the investigation was that we encountered a number of challenges regarding data quality and availability, which partially led to questions not being answered to the extent we had originally envisioned. These challenges do not solely exist at our site but arise generally from the common level of data documentation in everyday clinical practice and the prospective collection of data for research purposes, influenced by the degree of digitization of clinical data in German hospitals. We provide a detailed account of the 8 most impactful difficulties here to highlight the necessities, and we describe how we overcame some of them by identifying alternative solutions.

The first major limitation was the amount of data. While we had originally planned to export data from all 3 consortium hospitals, this was made impossible due to technical limitations as well as differing processes across the hospitals. As a consequence, this study is constrained to data from a single hospital. This resulted in a rather small sample size of 2986 patients, and this in turn excluded AI-based models that require much larger amounts of data [41]. Using data from several clinics would have increased the size of the dataset and provided a more representative sample of patients. It would also have enabled us to investigate how much the results differ between clinics. However, obtaining data from multiple clinics would have posed additional challenges due to expected heterogeneity in terms of patient population, prescribing practices, data quality, and other factors. An approach to jointly train generative models on data from multiple hospitals by using federated learning is described by Düsing et al [12].

A second substantial restriction resulted from the fact that several variables, which had been identified as highly important, were not present in the dataset. These included patient history information from which one could have derived the risk of individual resistance against certain antibiotics, such as origin, profession, travel history, and previous antibiotic prescriptions. Moreover, we lacked information on the occurrence of adverse effects, which was not even provided for severe cases. Consequently, our investigation could not address the identification of antibiotic therapies with minimal adverse effects.

Third, the suspected infection focus, which was highlighted by the doctors as one of the most relevant factors in the decision-making process for an antibiotic therapy, was not routinely documented. To still obtain a realistic prediction, patient diagnoses associated with the infection foci were used to infer the suspected infection focus for individual patients (refer to the preprocessing steps outlined in the Data subheading).

Fourth, the exact time of diagnosis was unknown in the sense that its temporal alignment was partially shifted when the documentation started after the first treatment. Hence, there is uncertainty regarding the patient’s condition at the time of diagnosis.

Fifth, regarding the effectiveness of an antibiotic therapy, the medical experts asserted that the clinical impression of a patient was given primacy over laboratory values. Still, no such data were documented. Thus, no direct quantitative measure was available to validate the effectiveness of the given therapies.

Sixth, even for those variables that were theoretically included in the dataset, there were many missing values. Thus, despite the ongoing digitization in hospitals and the consideration of retrospective data from more than 10 years, the final sample size of complete patient data was relatively small. Our decision not to impute missing values and to consider only complete cases further contributed to the small sample size and thus limited the feasibility assessment.

Seventh, particularly in the context of time series analyses, the longitudinal measurements are obtained at varying time intervals per patient and with differing frequencies among the measured values. The generation of the variable “minutes after diagnosis” (refer to the Data subheading) resulted in a data matrix for longitudinal values that was extremely sparse. This issue was addressed as described in the Statistical Analysis subheading.

Eighth, concerning the primary target variable, the empiric antibiotic therapy, we encountered high class imbalance (Figure 2). The 10 most frequently prescribed antibiotics accounted for more than 89% of all empiric antibiotics, with “Piperacillin/tazobactam” being the most frequently administered therapy, accounting for 54% (n=1765) of all prescriptions. This imbalance in prescriptions posed a major challenge in predicting outcomes for underrepresented classes and may have led to biased classification results [42]. We addressed this issue with a 2-step modeling approach as outlined in the Statistical Analysis subheading.

To evaluate our classification approach beyond the previously described dataset and to gain insights into the generalizability of our method, we sought to incorporate an additional dataset. The most suitable source for this purpose was the Medical Information Mart for Intensive Care, Fourth Version database [43], which contained health-related data from patients admitted to the ICU of the Beth Israel Deaconess Medical Center between 2008 and 2019. However, important information, such as the infection focus or procalcitonin measurements, were missing in the database. Further, we detected notable discrepancies between Germany and the United States of America with regard to the fundamental hospital procedures and guidelines for the management of sepsis, as well as the related data basis (eg, [44]), which disqualified the Medical Information Mart for Intensive Care, Fourth Version database as a solid basis for comparison within the presented feasibility study. Thus, to develop a targeted approach for common practice in Germany, we exclusively relied on the data described and emphasized the need for more and better data about the diagnosis and treatment of patients with sepsis.

### Learnings From Statistical Modeling

#### Cross-Sectional Classification

Despite the aforementioned challenges, we used various approaches to statistically model the physicians’ decisions for empiric antibiotic therapy. Table 3 shows the results of the CCMs (RF, GBC, SVC, and MLP) for the 3 exemplary antibiotics “Piperacillin/tazobactam,” “Ciprofloxacin,” and “Vancomycin.” The results of the remaining antibiotics are provided in MultimediaAppendices 2  3. Due to the outer loop of the nested CV, we obtain 10 predictions for different test sets; the tables present their averages.

**Table 3.** Average performance of the cross-sectional classification models (CCMs) across 10 folds of nested cross-validation (CV) for 3 selected antibiotics. Measures are calculated across both classification steps and thus represent cumulative performance.

Model and metric	Piperacillin/tazobactam	Ciprofloxacin	Vancomycin
RF^a^			
Sensitivity	0.506	0.117	0.125
Specificity	0.728	0.955	0.962
Precision	0.613	0.108	0.051
*F*_1_-score	0.552	0.110	0.069
GBC^b^			
Sensitivity	0.508	0.092	0.050
Specificity	0.642	0.985	0.969
Precision	0.546	0.117	0.050
*F*_1_-score	0.524	0.102	0.050
SVC^c^			
Sensitivity	0.524	0.095	0.120
Specificity	0.721	0.973	0.964
Precision	0.621	0.095	0.064
*F*_1_-score	0.564	0.095	0.072
MLP^d^			
Sensitivity	0.533	0.078	0.145
Specificity	0.677	0.973	0.949
Precision	0.594	0.108	0.052
*F*_1_-score	0.556	0.090	0.069

a RF: random forest.

b GBC: gradient boosting classifier.

c SVC: support vector classifier.

d MLP: multilayer perceptron.

It becomes immediately apparent that none of the models achieved high sensitivity, precision, or *F*_1_-score; consequently, the models are not reliable enough for use in clinical practice. For most antibiotics, the sensitivity is lower than 22%, and in many cases it is even lower than 10%. This low sensitivity comes along with a high specificity. The only exception is “Piperacillin/tazobactam,” for which all 4 classification models result in a sensitivity above 50% and a specificity above 60%. MLP achieved the highest sensitivity of 53.3%; this is accompanied by a medium-high specificity of 67.7%. Compared to all other antibiotic therapies, the performance of the 4 models for predicting “Piperacillin/tazobactam” is satisfactory: both sensitivity and specificity, and thus precision and *F*_1_-score, are moderately high. However, this performance is still insufficient for clinical implementation. Even worse results are obtained for “Ciprofloxacin” and “Vancomycin”: the highest sensitivity of 11.7% and 14.5% is achieved by RF and MLP, respectively.

#### Time Series Classification

The averaged results from time series classification across the 10 folds of the CV for the 3 antibiotics “Piperacillin/tazobactam,“ “Ciprofloxacin,” and “Vancomycin” are shown in Table 4. The results for the remaining antibiotics are provided in Multimedia Appendix 4. No improvement over CCM results could be achieved using time series data. *k*-NN with DTW performed worse than the CCMs as measured by *F*_1_-score. Even for “Piperacillin/tazobactam,” the *F*_1_-score was on average 12.3% points lower when using the TSCM instead of CCMs. The poor performance for “Ciprofloxacin” and “Vancomycin” is conspicuous. In the case of “Ciprofloxacin,” the sensitivity is low (2%), and the same holds for “Vancomycin”: not a single sample belonging to this class was correctly classified.

**Table 4.** Average performance of time series classification models (TSCMs).

Metric	Piperacillin/tazobactam	Ciprofloxacin	Vancomycin
Sensitivity	0.416	0.020	0.000
Specificity	0.572	0.991	0.952
Precision	0.457	0.125	0.000
*F*_1_-score	0.426	0.029	0.000

#### Baseline Model

To compare the results of the described CCM and TSCM against a simple baseline model, we predict the antibiotic therapy for all patients to be “Piperacillin/tazobactam” without further training of a statistical model. Naturally, for all antibiotics other than “Piperacillin/tazobactam,” the sensitivity, precision, and *F*_1_-score are zero, while the specificity is equal to one. The performance results for “Piperacillin/tazobactam” are shown in Table 5. As expected, the sensitivity equals one, while the specificity is zero. The *F*_1_-score for “Piperacillin/tazobactam” equals *2m / n + m*, where *n* is the total number of patients and *m* is the number of those belonging to this majority class. The value of 0.629 directly results from the imbalance of class sizes and is higher for the baseline model than when applying the CCM and TSCM. However, the *F*_1_-score for “no Piperacillin/tazobactam” equals zero. Precision is lower for the baseline model than for the CCM and similar to the TSCM. Consequently, when considering only the majority therapy, the complex modeling approaches are outperformed by a simple heuristic in terms of the *F*_1_-score. This is hardly surprising since the CCM and TSCM are trained to differentiate between a number of therapies rather than putting all emphasis on the majority class. The (moderate) success is reflected in the performance metrics of the other therapies.

**Table 5.** Performance of the baseline model for all antibiotics.

Metric	Piperacillin/tazobactam	Any other antibiotics
Sensitivity	1.000	0.000
Specificity	0.000	1.000
Precision	0.461	0.000
*F*_1_-score	0.629	0.000

## Discussion

### Summary

We embarked on an ambitious project to develop a CDSS aimed at recommending an appropriate initial antibiotic therapy for patients with sepsis. This CDSS was intended to be applicable immediately upon diagnosis and to provide recommendations regarding potential resistance and side effects. The system was to be developed using data science methods, drawing from data collected at 3 local hospitals.

However, the situation within the clinics revealed that various factors contributed to a relatively small dataset, comprising only 1194 complete cases, along with a substantial amount of unavailable information. Contemporary AI approaches typically require large datasets; therefore, a swift implementation using large language models, which may seem straightforward to an outsider, was not feasible in this context. Instead, we took on the challenge by combining meticulous information and data preparation with the adaptation of statistical learning methods. In fact, we achieved a satisfactory classification for one of the subquestions. Nonetheless, our project uncovered several limitations, and in this paper, we outline the key insights regarding the process.

To summarize, the process included the initial stages of knowledge acquisition, data preprocessing, overcoming data-related challenges, and constructing predictive models to classify the physician’s choice of empiric therapies. We have described each relevant step, examined the challenges involved, and presented potential models together with the results derived from the available data.

### Principal Findings: Exploration of Model Performance

Despite careful data preparation, modeling, and incorporation of clinical knowledge, the statistical models achieved only poor to moderate performance in predicting the physicians’ decision for empiric antibiotic therapy. Only the first step of the 2-step approach (ie, the classification of “Piperacillin/tazobactam” against all other therapies) succeeded to some extent. We identify several reasons that we suspect are contributing to this issue.

#### Sample Size

Good predictions require sufficient sample sizes. Our final dataset contained only 1194 samples in total. Of those, 550 belonged to the class “Piperacillin/tazobactam,” while all other class sizes ranged from 21 to 174. The comparably good performance in the first step of the classification procedure indicates that, all in all, our statistical models are appropriate as long as enough data are available. To put it another way, we suspect that the main reason for the poor overall predictive ability is the small sample and class sizes. Sepsis manifests in very different ways; to find similarities in patients’ health records, many more samples may be needed for analysis. To develop a reliable CDSS for clinical usage, future projects should be based on a large dataset containing several thousand patients, with particular consideration of class balance and complete data collection.

#### Broad-Spectrum Antibiotics

Notably, the most commonly prescribed therapy, “Piperacillin/tazobactam” (roughly 50%), along with several other documented treatments, are broad-spectrum antibiotics. This reflects how often this type of antibiotic is prescribed in clinical practice. In some cases, this antibiotic therapy is recommended by the guidelines; however, the high frequency of prescriptions also suggests a paucity of relevant information for a more targeted treatment decision. A retrospective evaluation of administered antibiotics by medical experts, under consideration of a patient’s disease course, could be valuable to identify a targeted and effective antibiotic therapy. In doing so, a CDSS could learn to predict the best antibiotic therapy, and its predictive performance would not be affected by erroneous data.

#### Class Imbalance

Not only was the sample size small, but the classes were also substantially different in size. The overrepresentation of “Piperacillin/tazobactam” led to a notable class imbalance. To deal with this, we decided to split the classification task into 2 steps. For the first step, the prediction of “Piperacillin/tazobactam,” no class rebalancing was needed, and we achieved comparably good results using CCMs. Still, these results are not yet suitable for clinical practice, highlighting the need for large, high-quality datasets, in particular for AI-based classifiers. In the second step, in contrast, we had to rebalance the classes as these were still unequally represented. Depending on the method used for rebalancing, useful information was removed (eg, when using random undersampling of the majority class [45]), samples were duplicated (eg, when using random oversampling of the minority class [45]), or synthetic data were added, potentially containing unrealistic or even wrong information (eg, when using the synthetic minority oversampling technique [46]). Consequently, the application of methods for class rebalancing comes with uncertainty. In particular, random oversampling of small classes might lead to model overfitting, resulting in a model’s inability to learn generalizable patterns in the data. This, in conjunction with the small sample size, is a possible explanation for the poor predictive ability of the models. As already mentioned, future projects should therefore attach great importance to reliable and balanced data collection. This is one of the most relevant conditions for success in achieving good model performance.

#### Observed Initial Treatments

To model a physician’s decision for an empiric antibiotic therapy, we used the first administered antibiotic contained in the dataset, regardless of whether the therapy was effective, because the dataset does not contain quantified information about the effectiveness (refer to Challenges from Data Availability subheading). However, there are indications that the first antibiotic was not necessarily the most effective one and could thus lead to inappropriate predictions: the data show that, due to constant reevaluation, the administered therapy was modified several times during one infection for many patients. As a consequence, our models describe prescribing habits rather than optimal treatment decisions. If appropriate information on effectiveness becomes available, the target variable could, however, be easily adapted.

#### Missing Information

As outlined in the Challenges from Data Availability subheading, relevant variables for an informed decision in favor of an antibiotic therapy were not included in the data. In particular, there were no variables that provided information needed to assess a patient’s risk of infection with resistant bacteria, such as origin, travel history, profession, and previous antibiotic prescriptions. In addition, a predictive model could benefit from information about hospital-specific antibiotic resistance patterns, especially if patient-specific risk factors are not available, to limit the eligible antibiotic therapies. This information is not included in our data; however, the development of a resistance observatory was a concurrent task of the “KINBIOTICS” consortium [13]. Without any of this information, the model may not be able to predict an antibiotic therapy that is consistent with the empiric therapy chosen by the physician. A detailed discussion with medical experts about risk factors, necessary information, and the medical process for deciding on an antibiotic therapy before data collection is crucial. Ideally, a CDSS is based on prospective data so that all necessary information can be documented and subsequently extracted. According to literature, the infection focus, information about previous hospitalizations or stays in nursing homes, travel history, and origin of a patient, comorbidities, and hospital-specific antibiotic resistance patterns are of utmost importance for choosing an adequate antibiotic therapy for ICU patients [47].

#### Uncertain Infection Focus and Time of Diagnosis

Medical experts regarded the infection focus as of high importance within the decision-making process for antibiotic therapy (refer to Requirements and Insights From Clinical Knowledge subheading). In the Data subheading, we explained how we derived the suspected focus. The alignment of confirmed and suspected focus, however, comes with uncertainty, and we consider possible errors to further affect the models’ performance. The use of retrospective discharge data to predict a hospitalization decision poses a limitation of our study, as it artificially inflates the feasibility of such a CDSS. A similar statement holds for the time point of sepsis diagnosis as derived by us. Our assumption that the time of sepsis diagnosis corresponds to the time of first administered antibiotic therapy will affect our models’ performance, as we use data for prediction that likely do not match the prescription. More precisely, our models might include values that were measured close to the time of administration of empiric antibiotics or even afterwards. This renders the models unsuitable for clinical application. This issue should be addressed in future studies by using prospective data that contains the exact time of diagnosis, as well as information on crucial variables between this time and the time of antibiotic administration.

#### Missing Data

Many classifiers are unable to deal with missing values. Thus, we reduced the dataset to complete cases by removing certain covariates and patients. This further limits the classification models’ interpretability and increases prediction uncertainty. Further, by removing laboratory values of high clinical importance, in particular procalcitonin, our models lack inflammatory markers.

In light of all the aforementioned limitations, the poor classification performance, especially in the second step of our approach, becomes comprehensible. The shortcomings highlight the need for large, high-quality data as a basis for a reliable CDSS for the individualized prediction of a targeted, effective antibiotic therapy for patients with sepsis. Future initiatives should place the highest priority on ensuring thorough and accurate data collection.

### Conclusions

#### Overview

Our work yields 2 principal take-home messages: first, the importance of interprofessional collaboration, and second, the need for high-quality data and improvements in data acquisition processes.

Regarding the first, interprofessional collaboration and the continuous exchange between medical experts and model constructors turned out to be crucial both in the preliminary stages of the project and during its entire progress. Such collaboration increases the chance of obtaining meaningful results as all stakeholders are aware of the requirements for data availability and quality. Many medical relationships require detailed discussion before they can be integrated into the data and modeling processes. Simultaneously, critical questions—often stemming from one party’s lack of expertise—can lead to new insights and perspectives.

Regarding the second, the results of the models highlight the critical need for comprehensive and large, high-quality data. In accordance with literature (eg, [47]), we particularly recommend the complete and correct collection of the following variables: time of diagnosis, administered antibiotics and their effectiveness, suspected infection focus at the time of antibiotic prescription, retrospectively confirmed infection focus, risk factors (eg, origin, profession, and travel history), and laboratory values (eg, procalcitonin, lactate, leukocytes, CRP, and IL-6). The performance of the model could be further enhanced by the incorporation of comorbidities, antibiotic allergies, and image data. At best, these values are available in electronic form, and a plausibility check is performed during data entry with the option for immediate clarification. The data should contain information on several thousand patients. We assume that an appropriate number of samples depends on the data quality.

#### Limitations and Recommendations: Data Documentation

Our dataset contains information on patients admitted to the ICU, where they are constantly monitored. Here, certain parameters are measured in an automated way, while the measurement of many other values has to be actively initiated and is carried out manually. The latter may lead to a lack of continuity in health records, and it is expected that data from less controlled units (such as non-ICU) are of even lower quality. This is a nationwide issue, beyond the study location of Bielefeld, partially caused by strict data protection regulations, including a rigorous interpretation of the European Union’s General Data Protection Regulation. While these rules ensure a high standard of privacy, they also contribute to comparatively slow progress in digital transformation, as reflected in the European Union’s Digital Economy and Society Index, where Germany is positioned in the middle range in digital public services and e-government readiness [48].

To improve the general documentation of health data, the optimization and expansion of digitization in German hospitals are of the highest importance. The implementation of the electronic patient record system (elektronische Patientenakte [ePA] [49]), which is currently being introduced in Germany, has the potential to offer great benefits for scientific analyses. Specifically, information regarding patients’ origin, travel history, professional background, and time of diagnosis could be made more readily accessible. Even information from previous inpatient or outpatient stays can potentially provide relevant information that would be available through the ePA. The benefit of the ePA for research purposes is only provided if access to the data is granted. The draft of the German “Forschungsdatengesetz” [50] aims at improving access to and use of data for research purposes, consequently contributing to an improvement in data availability. Moreover, a health data usage act (“Gesundheitsdatennutzungsgesetz“ [51]) was passed in March 2024 in Germany, with the objective of regulating the use of health data for research purposes in the public interest and for the data-based further development of the health care system. A further desirable future development would be the continued growth of extensive, high-quality datasets, such as those from the NFDI4Health initiative (Nationale Forschungsdateninfrastruktur für personenbezogene Gesundheitsdaten [52]), making small-scale, project-specific data collections unnecessary and/or serving as external data validation resources.

#### Limitations and Recommendations: Model Architecture

The context considered in the study led to the definition of 2 architectural elements that should be evaluated with caution and adapted to the specific context of application. These concern the 2-step approach and the choice of the performance measure for parameter optimization.

Regarding the presented 2-step approach, we observe that errors arising from misclassifications of samples in the first step are propagated to the second step. The low sensitivity and moderate specificity for “Piperacillin/tazobactam” indicate that a substantial number of patients are irretrievably misclassified in the first step: samples truly belonging to the class “Piperacillin/tazobactam” but classified as “no Piperacillin/tazobactam” can no longer be correctly identified in the second step, and vice versa for samples belonging to “no Piperacillin/tazobactam” that are predicted as “Piperacillin/tazobactam.” In this case, the 2-step procedure was motivated by the severe imbalance between class sizes, where upsampling or downsampling of all classes, including “Piperacillin/tazobactam,” would have been the less favorable option. In this sense, the model choice was meaningful. Nevertheless, the architecture should be tested for robustness when the approach is transferred to another dataset with different class distributions or potentially clearer class boundaries.

Regarding the performance measure, we aimed to simultaneously account for both false-positive and false-negative predictions, in line with our study consortium’s objective of predicting a targeted antibiotic with minimal side effects. Therefore, we placed particular emphasis on the *F*_1_-score. For the translation of this approach to other projects, we recommend close coordination with the practicing physicians and clinics and, if necessary, adaptation of the statistical optimization objective.

#### Future Directions

The logical next steps that arise from our project are to move beyond mere classification of therapies that were prescribed in practice to providing recommendations for therapies that are expected to have high effectiveness. To overcome the lack of a quantitative measure for administered antibiotic therapies, this may involve a data-driven assessment of the health status of patients with sepsis from time series of laboratory values and vital signs. This may provide a basis for the development of methods that can learn from retrospective data to predict the best possible therapy outcome for an individual patient with sepsis. The steps achieved and presented here will contribute to the development of an effective CDSS for antibiotic prescription in sepsis, ultimately improving the survival of patients with sepsis and helping to reduce antibiotic resistance.

## Supplementary material

10.2196/79929 Multimedia Appendix 1 Missing values in time-series data.

10.2196/79929 Multimedia Appendix 2 Additional results of cross-sectional classification models for the antibiotics “Ampicillin/sulbactam,” “Cefotaxime,” “Ceftriaxone,” and “Cefuroxime.”

10.2196/79929 Multimedia Appendix 3 Additional results of cross-sectional classification models for the antibiotics “Levofloxacin,” “Meropenem,” “Piperacillin/tazobactam-levofloxacin,” and “others.”

10.2196/79929 Multimedia Appendix 4 Additional results of time series classification models for the antibiotics “Ampicillin/sulbactam,” “Cefotaxime,” “Ceftriaxone,” “Cefuroxime,” “Levofloxacin,” “Meropenem,” “Piperacillin/tazobactam-levofloxacin,” and “others.”

## Abbreviations

AI: artificial intelligence
CCM: cross-sectional classification model
CDSS: clinical decision support system
CRP: C-reactive protein
CV: cross-validation
DTW: dynamic time warping
ePA: elektronische Patientenakte
GBC: gradient boosting classifier
*ICD-10*: *International Statistical Classification of Diseases, Tenth Revision*
ICU: intensive care unit
IL-6: interleukin 6
*k*-NN: *k*-nearest neighbor
MLP: multilayer perceptron
RF: random forest
SOFA: sequential organ failure assessment
SVC: support vector classifier
TSCM: time series classification model
WHO: World Health Organization
