# Supplementary material for: Development Process of a Clinical Decision Support System for Empiric Antibiotic Therapies in Patients With Sepsis: Case Study
Source: JMIR Med Inform. 2026 May 13;14:e79929. doi: 10.2196/79929 (PMC13170932; doi:10.2196/79929)
Supplement: Multimedia Appendix 2 [file medinform-v14-e79929-s002.pdf]

Table A.1: Average performance of the CCMs across ten folds of nested CV for considered antibiotics (part 1), in addition to Table 3.

		Ampicillin/Sulbactam	Cefotaxim	Ceftriaxon	Cefuroxim
RF					
	Sensitivity	0.126	0.135	0.212	0.117
	Specificity	0.924	0.919	0.949	0.955
	Precision	0.104	0.153	0.195	0.060
	F1-Score	0.109	0.133	0.165	0.075
GBC					
	Sensitivity	0.141	0.128	0.000	0.108
	Specificity	0.903	0.906	0.955	0.979
	Precision	0.108	0.107	0.000	0.095
	F1-Score	0.119	0.113	0.000	0.097
SVC					
	Sensitivity	0.165	0.108	0.173	0.067
	Specificity	0.901	0.898	0.934	0.961
	Precision	0.102	0.076	0.082	0.044
	F1-Score	0.122	0.087	0.104	0.052
MLP					
	Sensitivity	0.105	0.073	0.138	0.037
	Specificity	0.929	0.909	0.950	0.966
	Precision	0.101	0.070	0.087	0.083
	F1-Score	0.096	0.070	0.094	0.051
