# Supplementary material for: Development Process of a Clinical Decision Support System for Empiric Antibiotic Therapies in Patients With Sepsis: Case Study
Source: JMIR Med Inform. 2026 May 13;14:e79929. doi: 10.2196/79929 (PMC13170932; doi:10.2196/79929)
Supplement: Multimedia Appendix 3 [file medinform-v14-e79929-s003.pdf]

Table A.2: Average performance of the CCMs across ten folds of nested CV for considered antibiotics (part 2), in addition to Table 3.

		Levofloxacin	Meropenem	Piperacillin/Tazobactam-Levofloxacin	others
RF					
	Sensitivity	0.184	0.116	0.000	0.116
	Specificity	0.918	0.912	0.978	0.945
	Precision	0.112	0.099	0.000	0.265
	F1-Score	0.135	0.104	0.000	0.153
GBC					
	Sensitivity	0.083	0.143	0.033	0.118
	Specificity	0.939	0.907	0.986	0.919
	Precision	0.055	0.099	0.033	0.198
	F1-Score	0.061	0.112	0.033	0.142
SVC					
	Sensitivity	0.042	0.107	0.083	0.112
	Specificity	0.933	0.934	0.980	0.937
	Precision	0.033	0.109	0.045	0.249
	F1-Score	0.037	0.104	0.058	0.152
MLP					
	Sensitivity	0.081	0.156	0.000	0.157
	Specificity	0.937	0.934	0.974	0.925
	Precision	0.064	0.122	0.000	0.268
	F1-Score	0.070	0.132	0.000	0.191
