# Supplementary material for: Development Process of a Clinical Decision Support System for Empiric Antibiotic Therapies in Patients With Sepsis: Case Study
Source: JMIR Med Inform. 2026 May 13;14:e79929. doi: 10.2196/79929 (PMC13170932; doi:10.2196/79929)
Supplement: Multimedia Appendix 4 [file medinform-v14-e79929-s004.pdf]

Table A.3: Average performance of the TSCM across ten folds of nested CV for considered antibiotics, in addition to Table 4.

	Ampicillin/Sulbactam	Cefotaxim	Ceftriaxon	Cefuroxim
Sensitivity	0.053	0.047	0.048	0.058
Specificity	0.963	0.954	0.941	0.942
Precision	0.079	0.062	0.034	0.058
F1-Score	0.055	0.047	0.037	0.050

	Levofloxacin	Meropenem	Piperacillin/Tazobactam-Levofloxacin	others
Sensitivity	0.100	0.028	0.005	0.084
Specificity	0.885	0.977	0.919	0.917
Precision	0.006	0.065	0.025	0.131
F1-Score	0.011	0.038	0.009	0.093
