# Supplementary material for: Development Process of a Clinical Decision Support System for Empiric Antibiotic Therapies in Patients With Sepsis: Case Study
Source: JMIR Med Inform. 2026 May 13;14:e79929. doi: 10.2196/79929 (PMC13170932; doi:10.2196/79929)
Supplement: Multimedia Appendix 1 [file medinform-v14-e79929-s001.pdf]

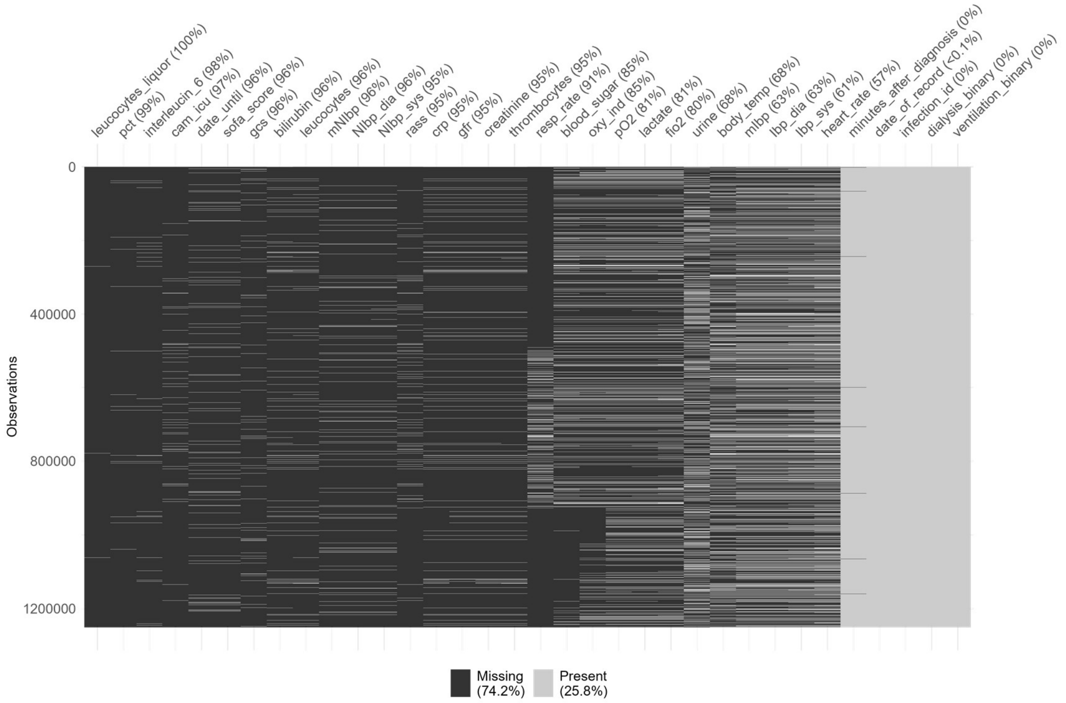

Figure A.1: Percentages and pattern of missing values in time series variables. Observations are shown in rows, variables in columns. Black areas are missing values while gray areas reflect available entries.
